# Associations between Lifestyle Changes and Adherence to COVID-19 Restrictions in Older Adults with Hypertension

**DOI:** 10.3390/ijerph19137853

**Published:** 2022-06-26

**Authors:** Marco D’Addario, Roberta Adorni, Patrizia Steca, Roberto Capelli, Francesco Zanatta, Francesco Fattirolli, Cristina Franzelli, Cristina Giannattasio, Andrea Greco

**Affiliations:** 1 Department of Psychology, University of Milano-Bicocca, 20126 Milan, Italy; marco.daddario@unimib.it (M.D.); patrizia.steca@unimib.it (P.S.); francesco.zanatta@unimib.it (F.Z.); 2 Department of Human and Social Sciences, University of Bergamo, 24129 Bergamo, Italy; roberto.capelli@guest.unibg.it (R.C.); andrea.greco@unibg.it (A.G.); 3 Department of Medical and Surgical Critical Care, Cardiac Rehabilitation Unit, University of Florence, 50139 Florence, Italy; francesco.fattirolli@unifi.it; 4 Azienda Ospedaliero-Universitaria Careggi, 50134 Florence, Italy; 5 Cardiac/Pulmonary Rehabilitation, ASST Gaetano Pini—CTO, 20122 Milan, Italy; cristina.franzelli@asst-pini-cto.it; 6 School of Medicine and Surgery, University of Milano-Bicocca, 20126 Milan, Italy; cristina.giannattasio@unimib.it; 7 Cardiology IV, “A. De Gasperis” Department, Ospedale Niguarda Ca’ Granda, 20162 Milan, Italy

**Keywords:** COVID-19, adherence to restrictions, lifestyle change, physical activity, diet, alcohol consumption, cigarette smoking, gender

## Abstract

COVID-19 has changed people’s routines and imposed new ways of living. This study investigated variations in lifestyles (namely, physical activity, diet, alcohol consumption, and cigarette smoking) between the prepandemic and the pandemic period in a sample of older adults with hypertension. Moreover, it investigated predictors of adherence to government restrictions during the first lockdown period, evidencing the role of relevant sociodemographic indicators and lifestyle changes. A sample of 105 older Italian adults (M_age = 70 years; SD = 5.83) with hypertension was enrolled from a previous longitudinal study and interviewed on the phone between May and August 2020. Updated information about sociodemographic indicators and lifestyle changes was collected. Adherence to restrictions was explored through several questions regarding compliance with home confinement, facemask use, and the observance of social distancing. Results evidenced that only 33% of the respondents abided by all the national restrictions. During the first pandemic peak, considerable changes in lifestyles occurred, particularly regarding physical activity, which diminished in 70% of the sample. Women, unemployed/retired people, and individuals who decreased their amount of physical activity reported higher adherence to rules. Maintaining a healthy lifestyle over time is essential for disease prevention. Therefore, it is essential to continue to inform the population about the importance of a healthy lifestyle, and it is necessary to provide guidelines to maintain and promote it even during housebound periods.

## 1. Introduction

The Severe Acute Respiratory Syndrome-Coronavirus-2 (better known as SARS-CoV-2 or COVID-19) started spreading worldwide at the end of 2019. Due to its contagiousness and lethality, on 11 March 2020, the World Health Organization declared the COVID-19 outbreak a global pandemic [1]. In March 2020, Italy was the European country with the highest number of confirmed cases and deaths, globally preceded only by China [2].

As for most countries worldwide, the Italian government put several measures to reduce the virus spread and safeguard people’s health, especially older adults and those with frailties [3]. During Phase 1 (proper lockdown; from 9 March to 4 May 2020; [4]), citizens were housebound: the only activities allowed were going to work, grocery shopping, and reaching hospitals for medical assistance. Companies that provided nonessential services and products had to allow their employees to telecommute. Universities, schools, restaurants, bars, theaters, cinemas, and clubs had to shut down. One meter of social distance was required, together with face masks [5]. Finally, a good cleaning and disinfecting routine were strongly recommended. During Phase 2 (after 4 May 2020; [6]), restrictions were reduced, although face masks and social distancing remained.

These restrictions, along with the fear of the virus, substantially impacted people’s psychological conditions: post-traumatic stress symptoms, insomnia, anxiety, and depression are among the most commonly reported [7].

The extent to which individuals have been conforming to stay-at-home orders and other protective measures and which are the best predictors of individuals’ adherence is less clear and remains a public health priority [8,9]. Prior research focused on the general population evidenced that women [8,9,10,11,12,13], people who live with others [14], and the unemployed [8,13,15] adhere more to behavioral restrictions than men, people living alone, and the employed.

Other studies have investigated lifestyle changes between the prepandemic and pandemic period. The lifestyle most compromised by the pandemic appears to be physical activity. Indeed, three available scoping reviews reported decreases in physical activity and increases in sedentary behaviors across several populations, including children [16,17], university students [18], and patients with a variety of medical conditions [16]. A very recent systematic review and meta-analysis [19] confirmed these results across age groups and independently of gender. Regarding the other lifestyles, a systematic review of longitudinal studies reported a worsening in the diet and increased alcohol consumption across different countries, age groups, and clinical and nonclinical populations [20]. However, the evidence of worsening of the diet is less consistent than that observed in the case of physical activity. Indeed, a second systematic review [21] reported an increase in adherence to the Mediterranean diet, but also a higher consumption of unhealthy food, such as snacks and sweets. Finally, available studies have reported increased addiction in smokers [22,23] but slight variation in smoking cessation/initiation [24].

It is worth noting that poor compliance with lifestyle recommendations during the pandemic period is documented in older individuals [25] and in patients with chronic conditions such as hypertension [26] and chronic coronary syndromes [27]. Lifestyle changes might increase anxiety, frustration, and stress and raise long-term disease risks. These issues are particularly relevant for those already suffering from chronic and predisposing conditions, such as hypertension and cardiovascular diseases [28].

A critical aspect in this regard is the fact that unhealthy behavior patterns are usually interrelated: people with unhealthy dietary routines practice little physical activity [29]; smokers who, despite the indications, do not quit report less adherence to pharmacological treatment [30]. Thus, adherence to health claims might be associated with adherence to behavioral restrictions related to the pandemic. In agreement with this observation, a recent study showed that healthy lifestyles were associated with higher adherence to protective behaviors and norms during the pandemic in a nonmedical sample of adults from Israel [31]. In the same line, unhealthy lifestyles—namely, drinking and smoking—have been identified as barriers to following government restrictions in people between 20 and 64 years in Denmark [32] and Japan [33].

To the best of our knowledge, no study has focused on the association between adherence to behavioral restrictions and lifestyle changes during the COVID outbreak in a frail population. Therefore, the overall purpose of the current study is to contribute to the very recent literature and focus on changes in daily habits caused by the pandemic—in terms of lifestyle and adherence to restrictions—in a sample of adults over 60 with hypertension, identifiable as frail due to their risk of developing severe forms of COVID-19 [34,35]. These individuals had taken part in a previous longitudinal study [36]. The longitudinal study offered the unique opportunity to monitor lifestyle changes before and during the pandemic and investigate significant predictors of adherence to COVID-19 restrictions.

The first aim was to describe lifestyle changes (physical activity, diet, alcohol consumption, and cigarette smoking) due to the COVID-19 outbreak. Data from the previous longitudinal study [36] made it possible to estimate lifestyle changes between the prepandemic and pandemic periods. A preliminary analysis was made to assess the stability of lifestyles in a “pre-covid period” covering 36 months, therefore testing the hypothesis that the changes in lifestyles were attributable to the COVID-19 pandemic. Based on previous pandemic-focused studies, we hypothesized that physical activity might significantly decrease, primarily because of restriction measures [19]. Furthermore, we expected that part of the sample might engage in “healthy” dietary patterns [21], whereas others in “unhealthy” ones [20]; alcohol intake may increase in some participants [20]; only a minor percentages of the sample are likely to start or stop smoking [24].

The second aim of the study was to investigate how lifestyle changes, along with the relevant sociodemographic indicators, might impact adherence to behavioral restrictions. As reported in a previous study on the same sample, only 33.3% of the respondents abided by all the national restrictions [37]. Based on the studies reported above, a series of hypotheses were made regarding the potential predictors of adherence. Regarding sociodemographic indicators, we hypothesized that women [8,9,10,11,12,13], people who live with others [14], and the unemployed [8,13,15] might report higher adherence to government restrictions. Moreover, recalling the interrelation between health behavior patterns [31,32,33], we hypothesized that people who worsened their lifestyles during the pandemic might be less willing to follow the rules.

Older individuals with noncommunicable diseases should pay close attention to the government’s restrictions, but they should also maintain a healthy lifestyle to safeguard themselves [34,35]. Therefore, it is essential to know how their lifestyle has changed in the pandemic period and if they have adhered sufficiently to the restrictions in order to implement targeted and effective communication strategies.

## 2. Materials and Methods

### 2.1. Participants and Procedure

For the present study, we recruited participants who took part in a previous longitudinal study.

The previous longitudinal study aimed to profile patients with essential arterial hypertension in various clinical, behavioral, and psychological variables [36]. The first recruitment occurred between February 2011 and May 2014 in a large hospital in Northern Italy. Inclusion criteria were an age of 30 to 75 years, a diagnosis of essential arterial hypertension (elevated blood pressure values, including systolic blood pressure (SBP) ≥ 140 mmHg or diastolic blood pressure (DBP)) ≥ 90 mmHg), and sufficient knowledge of Italian. The exclusion criterion was the presence of cognitive impairment due to a diagnosed neurological disease. A trained researcher collected data using self-report questionnaires.

To carry out the present study, we selected a subsample of patients over 60 from the previous longitudinal study. The aim was to focus on a “frail” population segment, i.e., at risk of contracting a severe form of COVID-19 [38].

A total of 232 patients were selected and contacted by telephone. Of the 232 patients contacted, 127 did not participate in the study. Among the 127 nonparticipating patients, 104 did not answer the phone call or had died, while 23 refused to participate. The remaining 105 patients participated in the study. The 23 patients who refused to participate did not differ significantly from the 105 participants by gender (χ^2^(1,128) = 0.216; *p* = 0.642) or education (χ^2^(1,128) = 0.049; *p* = 0.824). However, the 23 patients who refused to participate differed significantly from the 105 participants in age (U = 522.000; z = −4.260; *p* < 0.001), being on average older (77 years; SD = 6.66) than the participants (70 years; SD = 5.83).

The 105 participants had a mean age of 70 years (SD = 5.83) and were primarily men (60.6%). The high percentage of men in our sample reflects the higher incidence of cardiovascular disease in men compared with women [39]. Most of the participants were retired (73.1%), had a high school diploma (53.8%), lived with others (87.5%), and had suffered from hypertension for more than ten years (76.0%).

A trained interviewer administered the participants a structured telephone interview between the end of May and the beginning of August 2020. The interviewer collected information about the updated sociodemographic characteristics, health conditions, lifestyles, and adherence to the indications and restrictions imposed by the Italian government to limit the spread of COVID-19 (Appendix A). Data from the previous longitudinal study (collected at baseline and 6, 12, 24, and 36 months after baseline) were used in the present study to evaluate sociodemographic indicators and lifestyle changes.

The Ethical Committee of the authors’ university approved both the previous longitudinal study and the present study. All participants received written information about the study and signed a consent form to participate.

The sample size’s adequacy was established by resorting to Power Analysis [40] using the software G*Power Version 3.1.9.7 [41]. We computed the achieved power, given α, sample size, and effect size of the resulting model.

### 2.2. Demographic and Clinical Indicators

The first part of the telephone interview was about sociodemographic and clinical aspects. Besides age, gender, and education, which were already explored at the baseline of the longitudinal study, participants were asked to indicate who they currently lived with (alone vs. with others) and to define their working status (employed vs. unemployed/retired). Further questions investigated patients’ general health, for example, if they went through cardiovascular complications or faced other specific diseases. Additionally, participants were asked if they had contracted COVID-19 and, if so, the severity of the disease. The same question was asked about their loved ones.

### 2.3. Physical Activity

In the previous longitudinal study, physical activity was measured using the Rapid Assessment of Physical Activity Questionnaire-1 (RAPA-1; [42]). According to the American Heart Association [43], this tool is one of the most common and valid in investigating physical activity. It is a seven-item questionnaire that uses dichotomous queries to assess the amount of aerobic physical exercise done by the participant and assigns a final score from 1 (i.e., absence of physical activity) to 7 (i.e., vigorous physical activities). Therefore, the higher the final score, the healthier the amount of physical activity. In order to use data for the present study, the final score of each participant was recoded on three levels as follows: “Insufficient” for little to no physical activity; “Mediocre” for some light physical activity every week, or some moderate physical activity, but not every week; “Adequate,” for at least 20 min of vigorous physical activity three or more days per week every week.

During the telephone interview, participants reported their physical activity routines in a narrative way, and the interviewer classified answers following the categorization just described.

### 2.4. Diet

In the previous longitudinal study, the Italian version of the Mediterranean Diet Scale was employed to evaluate dietary routines [44,45]. The participant is asked to report the frequency of consumption of eight different food types through a 6-point Likert scale, where 1 means “Never” and 6 means “More than three times per day.” The eight categories considered in the questionnaire are vegetables, fruits, whole grains, wine, fish, red or processed meat, legumes, and olive oil. Following Trichopoulou et al. [45], a dichotomous variable was created to recode every response, where 0 indicated unhealthy and 1 healthy consumption of the specific food category. The sum of the recoded responses gives the final score. Dietary routines were classified into three categories: 0 = “inadequate” (total score ≤ 4); 2 = “good” (total score between 5 and 6); 3 = “excellent” (total score > 6). Internal consistencies and construct validity of the MDS have been demonstrated by previous research [45,46].

During the telephone interview, participants reported their diet routines in a narrative way, and the interviewer classified answers following the categorization just described.

### 2.5. Alcohol Consumption

In the previous longitudinal study, the participant reported their consumption of beer, wine, and spirits, following previous studies’ classification [47]. The final score was calculated as the mean of the three scores. Three levels were computed: 1 = “A teetotaler (never drinks alcohol—wine, beer, or spirits)”, 2 = “An occasional drinker (drinks on occasion, not every day)”, and 3 = “A regular drinker (drinks alcohol daily).”

During the telephone interview, participants’ alcohol intake was assessed and classified following the same criteria.

### 2.6. Cigarette Smoking

In the previous longitudinal study, according to previous research [48], a single question was asked to assess participants’ smoking behavior: “Do you currently smoke?”. We categorized the answers into three levels: 1 = smokers or ex-smokers who had quit less than a year before the assessment; 2 = ex-smokers who had quit more than a year before; 3 = those who had never smoked in their lives. This criterion was chosen based on previous literature on the topic [49], showing that the physical and psychological side effects (e.g., nicotine withdrawal) can occur for 12 months after quitting.

During this study, the same question and classification criterion was used.

### 2.7. Adherence to Behavioral Indications and National Restrictions

The interviewer asked a series of questions to explore adherence to national restrictions during the first lockdown phase (9 March to 4 May) and the subsequent phase (starting from 4 May). The interviewer asked how often and why the respondents left the house, if they went out alone, if they always wore a mask, and if they respected the distance of one meter from other people. The interviewer also asked what behaviors they adopted inside their home, if they received guests, if they always wore a facemask, and if they respected the distance of one meter from other people when they received someone at home.

For each behavior, we created a dichotomous variable that identified adherence (score = 1) or nonadherence (score = 0) to the government’s indications. Then, we created a dichotomous variable that synthesized “adherence behaviors”. Considering the importance of maximally adhering to behavioral restrictions, participants who had complied with all the restrictions were classified as adherents (score = 1). Participants who did not comply with at least one of the restrictions were classified as nonadherents (score = 0).

### 2.8. Statistical Analyses

Data collected in the previous longitudinal study (both at the baseline and at the four successive time points) were used to investigate the stability of lifestyles in the prepandemic period. Five Repeated Measures Analyses of Variance (RMANOVA) were carried out. The mean score of each behavior was the dependent variable, whereas time was the independent variable (5 levels: baseline, 6-, 12-, 24-, and 36-months). Post-hoc LSD tests were used for multiple comparisons of means.

A binomial logistic regression analysis was performed, with the summary variable “adherence behaviors” as the dependent variable (2 levels: adherent, nonadherent) and the relevant sociodemographic indicators (gender, living condition, and working status) and lifestyle changes as the categorical independent variables.

A *p*-value ≤ 0.05 was considered to indicate a statistically significant test for all analyses. Data analyses were performed using the IBM SPSS Statistics for Windows, version 26.0 (IBM Corp., Armonk, NY, USA) and Jamovi (Version 2.2.5) (The jamovi project, 2021, retrieved from https://www.jamovi.org, accessed on 17 May 2022).

## 3. Results

### 3.1. Preliminary Analyses: Lifestyles Stability during the Prepandemic Period

The analyses performed considering the mean score of each lifestyle as the dependent variable and time as the independent variable showed that time had no significant effect on the lifestyles (see Appendix B). The only significant difference occurred between the baseline and the 24-month time-point and regarded alcohol consumption (*p* = 0.01). The means on the other time points were steady. Therefore, the variation was considered irrelevant for the present study.

These results show that throughout the previous longitudinal study—i.e., over 36 months—lifestyles did not change. This result suggests that lifestyles can be considered participants’ stable habits in the prepandemic period, from the beginning of the original longitudinal study—about ten years ago—up to the period immediately preceding the pandemic. For this reason, data collected at the baseline of the longitudinal study were used in the following analyses.

The change between baseline—indicative of the prepandemic—and the present study—indicative of the pandemic period—was investigated for each lifestyle variable. A delta value was calculated by subtracting the value of the lifestyle in the pandemic period to that of the prepandemic period (e.g., Δ Physical-Activity = Physical-Activity pandemic—Physical-Activity prepandemic).

The results of each lifestyle were recoded into three levels:

−1 = “Decreased”, 0 = “Maintained”, and 1 = “Increased”

A “decrease” in physical activity and in a healthy diet indicates a worsening behavior. A “decrease” in alcohol consumption and smoking indicates an improving behavior.

The four variables were considered for evaluating the change in lifestyles in the pandemic period and the role of this change in predicting adherence to government restrictions.

### 3.2. Participants’ Health Condition

Overall, 92.4% of the participants reported having good general health, even though 23 underwent some operation or treatment that required hospitalization between the last assessment of the longitudinal study (about five years before) and the telephone interview. Fourteen had cardiovascular complications, six oncological, and three pneumatological ones. Most participants reported no COVID-19 contagion (83.7%) at the time of the telephone interview, but the remaining 16.3% declared they did not undergo any screening tests and, thus, did not receive a diagnosis. Most of the participants (75.5%) said they did not know someone diagnosed with COVID-19, whereas the remaining 24.5% did.

### 3.3. Lifestyles Change

**Figure 1:**
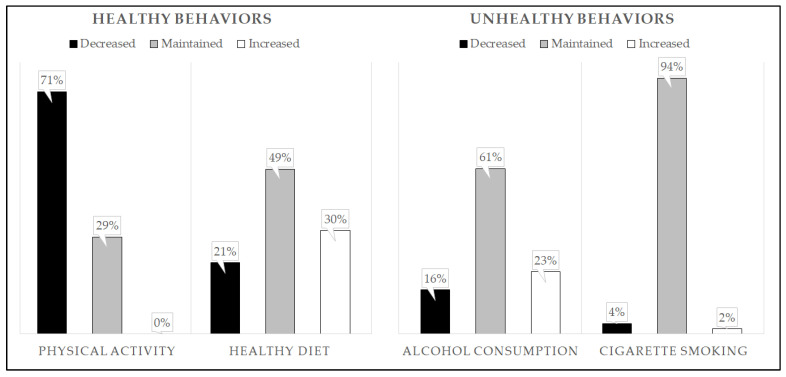
Percentage of participants who decreased, maintained, or increased their behavior concerning the four lifestyles between the prepandemic and pandemic period. A “decrease” in physical activity and a healthy diet indicates a worsening behavior, whereas a “decrease” in alcohol consumption and smoking indicates an improving behavior. An “increase” in physical activity and a healthy diet indicates improved behavior, whereas an “increase” in alcohol consumption and smoking indicates a worsening behavior.

None of the participants increased their physical activity between the prepandemic and the pandemic periods. 71.4% of the sample reduced their amount of exercise, while 28.6% kept the same. Regarding diet and alcohol consumption, about half of the sample maintained the prepandemic behavior, while a small percentage worsened or improved their behavior. Finally, almost all of the sample (94.3%) did not change their smoking behavior. Figure 1 details these results.

### 3.4. Adherence to Government Restrictions

Overall, 33.3% of the sample adhered to all the indications and restrictions imposed by the Italian government, whereas the remaining 66.7% failed to follow at least one rule. Concerning Phase 1, the specific behavior that was least respected was about meeting others when outside (adherence = 66.7%). However, the use of facemasks and the observance of one-meter social distancing were employed by almost the whole sample (adherence = 97.2%) when leaving home. During Phase 2, respondents maintained adherent behavior regarding social distancing and facemask use when leaving home (adherence = 96.2%). However, when meeting their acquaintances, respondents tended to respect social distancing less and not wear a facemask (adherence = 56.7%). Appendix C synthetizes these results.

### 3.5. Prediction of Adherence to Restrictions

The binomial logistic regression results indicated that the full model was statistically significant, χ^2^(10, N = 105) = 27.2, *p* = 0.002. Thus, the model was able to distinguish between respondents who were adherent to the behavioral restrictions vs. those who were not. The model explained between 23% (Cox and Snell’s R^2^) and 32% (Nagelkerke’s R^2^) of the variance in adherence to restrictions.

As illustrated in Table 1, three independent variables (gender, occupation, and change in physical activity) made a unique, statistically significant contribution to the model.

The analysis suggested that men were less prone to abide by the rules than women (B = 1.06, SE = 0.50, *p* = 0.036; achieved power = 0.90). The odds ratio indicated that men were three times more likely to disregard indications than women (OR = 2.88). Participants who worked tended to be less adherent than nonworking ones (B = −1.34, SE = 0.61, *p* = 0.027; achieved power = 0.97). The odds ratio showed that the former were almost four times more likely to report nonadherent behaviors than the latter (OR = 3.84). Finally, individuals who decreased their physical activity between the prepandemic and the pandemic period were more likely to follow national indications (B = 1.36, SE = 0.59, *p* = 0.022; achieved power = 0.97). The odds ratio showed that participants who did less physical activity during the pandemic than during the prepandemic period were four times more likely to abide by the rules than those who did not change their motor routines (OR = 3.90).

## 4. Discussion

The present study aimed to explore lifestyle changes from the prepandemic period to the pandemic in a sample of older Italian adults with hypertension. Moreover, it investigated predictors of adherence to restrictions, focusing on the role of the relevant sociodemographic indicators and lifestyle changes.

Regarding the study’s first aim, we identified several lifestyle changes during the COVID-19 pandemic. These changes are relevant considering the strong stability highlighted in the prepandemic period. Indeed, preliminary analysis showed that lifestyles were stable over the 36 months prior to the pandemic.

The behavior that varied most was physical activity. None of the participants increased their amount of exercise, and more than 70% decreased it. The effects of the pandemic and the national restrictions on this lifestyle have been investigated by many authors, who underlined a significant decrease in training routines and the number of steps per day during the pandemic period across different segments of the population [16,17,18,19]. This decrease is particularly significant in older adults in contrast to children, adolescents, and (young) adults [19]. These results are not surprising: a massive reduction in exercise can be ascribed to the impossibility of leaving the house. Moreover, as shown in other research, changing and maintaining good physical activity is one of the most challenging goals in patients affected by cardiovascular diseases [50]. Additionally, considering the mean age of our participants, a lack of practical knowledge about internet tools [51,52] could have further undermined physical activity routines. Maintaining a physically active lifestyle is crucial to preventing various diseases. Therefore, it is essential to provide people with the correct information about its role and guidelines to maintain and promote it even during housebound periods, favoring access to digital alternatives and technological know-how [19].

The pandemic led people to change their eating routines. Two different behaviors were observed: about 31% of the sample paid greater attention to their food quality, while 21% of the participants worsened their dietary routines. These findings are in line with the ones pointed out by Górnicka et al. [53], who found that during the COVID-19 pandemic, part of the study sample engaged in “healthy” dietary patterns and others in “unhealthy” ones. Further studies are needed to clarify which elements played a role in directing these behaviors. On the one hand, greater availability of time could have led people to pay more attention to grocery shopping and to devote themselves to preparing food, as argued in a recent report published by the European Institute of Innovation & Technology [54]. On the other hand, psychological distress and social isolation might be the causes of worsened dietary behavior [55].

Similar changes were found for alcohol consumption. As highlighted in another study [23], some participants increased alcohol intake (23%), while others reduced it (16%). Again, psychological distress resulting from the pandemic may be responsible for increased alcohol consumption, as Rodriguez et al. [56] suggested. Conversely, the closure of bars, pubs, and restaurants might have facilitated a reduction in some of the participants.

Smoking encountered only marginal changes between the two time-points analyzed. The slight decrease in smoking behaviors could be ascribed to the fear of developing more severe respiratory distress from COVID-19 [24]. On the other hand, its increase was mainly related to boredom during lockdown measures [23].

Regarding the second aim of the present work, it is worth noting that, as reported in a previous paper [37], only 33% of the sample abided by all the limitations imposed by the Italian government. This low level of adherence could be explained considering the strictness of the evaluation we carried out. Indeed, we classified as “adherent” the participants who respected all the restrictions, with no exceptions. Previous studies showed heterogeneous results: in some cases, restrictions were followed by 35% to 45% of the sample, whereas elsewhere, adherence reached almost 70% to 90% [10,12,13,14]. The Webster et al. [57] review identified factors associated with adherence to quarantine during infectious disease outbreaks and pointed out that the adherence percentages reported by the previous studies ranged from 0 to 93%. This variation is partly due to the different methods employed during the assessments: sometimes, a single behavior was considered, or the overall score was not recoded in a dichotomous variable. The present study investigated adherence behaviors in-depth and provided a comprehensive assessment while strictly following the indications promoted by the Italian government. Importantly, as already discussed in a previous work [37], our results show that difficulty adhering to all the restrictions is also true for the older adults with a chronic clinical condition—that is, people at higher risk for virus contraction’s worst consequences.

The analysis exploring the predictors of adherence behaviors showed that women and unemployed/retired participants reported higher adherence to rules. Differences concerning gender might result from a higher inclination to risk-taking conduct by men [58]. Similar results were reported by other authors who investigated behaviors during the COVID-19 pandemic [8,9,10,11,12,13]. Regarding working status, the fact that employed people were more likely to leave the house and meet with other individuals might have increased the risk of disregarding national rules. In line with this hypothesis, previous studies showed that situational variables (e.g., opportunity to violate norms) strongly affected participants’ compliance with social distancing measures [8,13,15]. Unlike the initial hypothesis, the living condition was not a significant predictor. Almost 90% of the participants lived with others; therefore, the low heterogeneity of our sample could have affected the results.

Changes in physical activity between the prepandemic period and the pandemic also showed a significant role in predicting adherence. The most important insight provided by the present work is that participants who did not decrease their amount of physical activity reported a significantly lower level of adherence to national restrictions. The World Health Organization promoted physical activity during housebound periods of the COVID-19 pandemic [54]. It is, therefore, necessary to further investigate the relationship between changes in physical activity and adherence to rules, regardless of the causal direction between the two factors. Several aspects are involved. First of all, participants may have decided to disobey certain restrictions (e.g., home confinement) to maintain their exercise routines and social relationships. This aspect would be particularly true for older adults, where an activity such as going for a walk also represents an opportunity to nurture social life [59]: this is even more essential during the pandemic period. In line with this hypothesis, the lowest levels of adherence were connected with meeting acquaintances in our sample. In addition, as argued before, older adults may encounter troubles in adopting internet-based solutions for staying physically active. This aspect could further push them to undertake outdoor exercises, disregarding rules.

Regardless of the causal direction between the two factors, our results suggest that a lot needs to be done to promote physical activity and adherence to the rules. Jiménez-Pavón et al. [60], as well as Füzéki et al. [61], showed how both young and older adults could easily maintain training routines during housebound periods. They might perform aerobic, resistance, balance, coordination, and mobility exercises even in small places without proper devices. Governments should favor such activities, for example, through bonuses on home equipment. Considering inexperience with new technologies, informative booklets with recommendations and examples of exercises should be provided to older people.

The present work has some limitations. First, there have been differences in collecting data between the prepandemic longitudinal study and the present study. The participants completed self-report questionnaires in the previous longitudinal study that provided a schematic, uniform description of their characteristics. Conversely, in the present study, information was acquired narratively; therefore, it is more in-depth but less uniform. We chose this method because self-report questionnaires would have been complicated given the government’s restriction measures. Indeed, older adults are usually unfamiliar with the internet and online surveys. Thus, the telephone interview represented the best way to reach this specific category of people. Second, the present study merely relies on a self-report method, presenting inferential limitations (e.g., answers about adherence behaviors could have been influenced by social desirability bias; [62]). This method offers significant advantages; for example, it can be applied simply in clinical research settings and is cost-effective. A recent study also showed overlap between self-reported and actual behaviors during the COVID-19 pandemic [63]. However, it would be advisable in future studies to supplement the self-report assessment with objective behavioral measures, notably concerning physical activity [64]. Finally, the study’s replicability is limited to a specific category of people, namely, older adults with hypertension. Although its replicability is limited, the results of this study can provide important insights about a specific category of people only marginally considered in recent literature about the pandemic. Due to their frail condition, older adults with noncommunicable diseases are more likely to develop severe forms of COVID-19 and should therefore be safeguarded the most.

Despite limitations, several strengths stand out. The possibility to compare current behaviors with longitudinal information on the prepandemic period is a distinctive strength of this study. It demonstrates the consequences of the ongoing situation on people’s lives and provides the opportunity to analyze predictors of adherence behavior. Indeed, the present study is the first to explore multiple predictors of adherence to COVID-19-related restrictions, investigating sociodemographic variables and lifestyles. As already underlined, the extent to which individuals—particularly older patients with chronic diseases—have been adhering to protective health measures and which are the best predictors of individuals’ adherence remains a public health priority [8,9].

## 5. Conclusions

Our results confirm that the difficulty of adhering to protective health measures also involves the elderly and clinically frail population. This evidence is essential considering that this population segment has a high risk of contracting severe forms of COVID-19.

Moreover, they confirm that sociodemographic characteristics, particularly gender and working status, influence adherence to protective health measures.

Our findings also contribute to the current knowledge about the impact of the COVID-19 pandemic on daily lifestyles. The most relevant finding is that physical activity is the lifestyle most compromised by the pandemic and the only one significantly associated with adherence to the pandemic containment restrictions. Maintaining a physically active lifestyle is essential for disease prevention. Therefore, it is necessary to provide guidelines to maintain and promote it even during housebound periods. There are numerous recommendations on how young and older adults could easily maintain training routines during housebound periods [60,61,65,66].

A valuable strategy could be to foster access to digital alternatives and technological know-how. Indeed, Parker et al. [67] found that users of digital platforms were more likely to adhere to PA guidelines than nonusers during the pandemic.

## Figures and Tables

**Table 1:** Results of the binomial logistic regression analysis.

Predictor	B	SE	*p*	OR	95% Confidence Interval	
					Lower	Upper
Gender	1.06	0.50	0.036	2.88	0.13	0.93
Living condition	1.29	0.90	0.154	3.62	0.62	21.26
Occupation	1.34	0.61	0.027	3.84	1.16	12.64
Change in physical activity						
Decreased—stable	1.36	0.59	0.022	3.90	1.22	12.49
Change in dietary routines						
Decreased—stable	1.09	0.62	0.080	2.98	0.88	10.16
Increased—stable	0.17	0.59	0.772	1.19	0.37	3.76
Change in alcohol consumption						
Decreased—stable	−1.21	0.74	0.104	0.30	0.07	1.28
Increased—stable	−0.22	0.62	0.719	0.80	0.24	2.70
Change in smoking behaviors						
Decreased—stable	−17.19	1975.65	0.993	0.00	0.00	Inf
Increased—stable	−17.44	2443.74	0.994	0.00	0.00	Inf

Note. B = Estimates—it represents the log odds of “adherent” (score = 1) and “nonadherent” (score = 0) participants. SE = Standard Error. *p* = *p* value. OR = Odds Ratio. Inf = Infinite

## Data Availability

The data presented in this study are available on request from the corresponding author. The data are not publicly available due to privacy and ethical restrictions.

## Appendix A

The following table illustrates the script used for the telephone interview.

**Domain of Investigation**	**Specific Questions**
General health information	How is your health in general? (Coded on a 5-point Likert scale) Have you developed any other cardiovascular or other diseases since we last spoke? (Interviewer annotated which one and when)
Lifestyle changes	Have you struggled to maintain healthy habits in the past few months? (Interviewer collected information about physical activity, diet, alcohol consumption, and smoke)
COVID information	Have you ever tested positive for the virus? If so, the interviewer asked how he/she found out, if he/she had been hospitalized and for how long, and how long the illness had lasted overall. Has anyone close to you tested positive for the virus? Who?
Adherence to national restrictions during phase 1 (from 9 March to 4 May 2020)	If you went out, how often did you do that, and why? If you went out, did you always wear a facemask, and did you always keep a distance of at least 1 m from other people? If you went out, were you alone? If you went out for exercise, did you go far from home? If you went out, did you meet anyone besides members of your household? If you stayed at home, did you meet anyone besides members of your household? If you stayed at home and met other people, did you always wear a facemask and maintain a minimum distance of 1 m from them?
Adherence to national restrictions during phase 2 (after 4 May 2020)	When you met someone, did you always wear a mask, and keep a distance of at least 1 m from other people? When you went out, did you always wear a mask, and keep a distance of at least 1 m from other people?

## Appendix B

The following table reports the results of the RMANOVAs analyzing lifestyle changes over the prepandemic period (baseline, 6-, 12-, 24-, and 36-months’ time-points of the previous longitudinal study). The only significant difference occurred between the baseline and the 24-month time-point and regarded alcohol consumption (*p* < 0.05). The means on the other time points were steady. Therefore, the variation was considered irrelevant for the present study.

**Lifestyle**	**Baseline Mean (SD)**	**6-Months Mean (SD)**	**12-Months Mean (SD)**	**24-Months Mean (SD)**	**36-Months Mean (SD)**	**F**	***p***
*Physical Activity*	4.71 (1.80)	4.58 (1.50)	4.84 (1.49)	4.71 (1.74)	4.76 (2.00)	0.38	0.79
*Diet*	1.31 (0.79)	1.38 (0.62)	1.42 (0.74)	1.26 (0.70)	1.31 (0.81)	0.93	0.43
*Alcohol Consumption*	2.15 (0.73)	2.25 (0.70)	2.16 (0.69)	2.31 (0.61)	2.07 (0.80)	3.43	0.01
*Cigarette Smoking*	4.16 (1.30)	4.15 (1.30)	4.15 (1.30)	4.24 (1.15)	4.36 (1.00)	3.00	0.06

## Appendix C

The following figure reports the percentage of participants who adhered to or did not adhere to the indications and restrictions imposed by the Italian government.
